# A Narrative Review on the Clinical Utility of Electrical Impedance Spectroscopy for Diagnosing High-Grade Cervical Intraepithelial Neoplasia

**DOI:** 10.7759/cureus.61784

**Published:** 2024-06-06

**Authors:** Georgios P Panagakis, Nikolaos Machairiotis, Maria Tsiriva, Charalampos Theofanakis, Paraskevi Tsetsa, Athanasios G Pantelis, Nikolaos Thomakos, Alexandros Rodolakis, Dimitrios Haidopoulos

**Affiliations:** 1 Department of Obstetrics and Gynecology, Alexandra General Hospital, National and Kapodistrian University of Athens, Athens, GRC; 2 Department of Obstetrics and Gynecology, Attiko University Hospital, National and Kapodistrian University of Athens, Athens, GRC; 3 Surgeon, Obesity and Metabolic Disorders Department, Athens Medical Group, Psychiko Clinic, Athens, GRC

**Keywords:** colposcopy, cervical cancer diagnosis, cervical cancer prevention, cervical intraepithelial neoplasia, zedscan, electrical impedance spectroscopy

## Abstract

Colposcopy constitutes a pivotal step in the diagnosis and management of cervical intraepithelial neoplasia; nevertheless, the method has several inherent and external limitations. Electrical impedance spectroscopic (EIS) has been among the adjuncts that have been developed to increase the diagnostic accuracy of colposcopy. EIS is based on the principle that the trajectory of electrical current alters depending on the consistency of the tissues. In the present study, we investigate the diagnostic accuracy and clinical utility of EIS by means of searching the available evidence. Our search yielded 17 articles during the period 2005-2023. Subsequently, we focused on the performance metrics of the included studies. The general concept is that EIS, in combination with colposcopy, is a method with increased sensitivity and specificity in detecting high-grade cervical intraepithelial neoplasia as compared to colposcopy alone. However, we documented a heterogeneous distribution of these and other metrics, including the positive predictive value, the negative predictive value, and the area under the receiver operating characteristic curve (AUC). Additionally, we located potential confounders that might hamper the measurements of EIS and, as such, warrant further investigation in future research. We conclude that future studies should be directed towards randomized multicentric trials, whereas the advent of artificial intelligence might improve the diagnostic accuracy of the method by helping incorporate a large amount of data.

## Introduction and background

Cervical cancer remains a significant health concern despite widespread screening, prompt diagnostic modalities, and effective preventive measures, including HPV vaccination programs [1]. Its burden yields more than 13,000 new cases and 4000 deaths annually in the United States [2], whereas these numbers are even higher in certain regions of the world (i.e., Eastern Mediterranean) and particularly low- to middle-income countries, classifying cervical cancer as the fourth most common cancer in females globally, with an annual incidence of 660,000 new cases and 350,000 deaths worldwide [3]. Early detection and effective treatment of high-grade cervical dysplasia are pivotal in reducing morbidity and mortality. Conventional screening with Pap smears, HPV-DNA testing, colposcopy, and visual inspection with acetic acid (VIA) has proven effectiveness but also bears several limitations, both inherent (sensitivity, specificity, positive and negative predictive value) and external (training and experience of the examiner, prevalence of cervical cancer in the particular population, number of biopsies performed, etc.) [4,5]. This has led to the development of various device-assisted screening methods, including the AV magnivisualizer, Point of Care Tampon (POCkeT), TruScreen (Sydney, Australia), ZedScan, CervAstra (a computational pathology platform), diSYSmap (computer-aided colposcopy with cervical mapping), and LuViva (hyperspectral imaging spectroscopy), each one applying different principles in order to increase the diagnostic acuity of cervical neoplasia at a preclinical stage [6,7].

ZedScan is a diagnostic tool that utilizes electrical impedance spectroscopy (EIS) and is based on the principle that electrical current follows different pathways depending on its frequency but also on the structure of the tissue, which consists of cells, intracellular and extracellular fluid [8]. The hypothesis is that tissue conductivity (and consequently impedance) is differentiated between tissue types, as is the case with normal, precancerous, and malignant lesions. A recent review identified 51 articles that covered 16 different cancer types (including high-grade cervical intraepithelial neoplasia (HG-CIN), melanoma, non-melanomatous skin cancer, and oral squamous cell carcinoma), for which EIS has been applied as a diagnostic modality [8]. ZedScan is designed to work in conjunction with colposcopy, providing real-time, objective data to guide biopsies and improve the diagnostic accuracy of the method. The enhanced precision of ZedScan may lead to better-targeted biopsies, reducing unnecessary invasive procedures (false positive rate), conversely ensuring that high-grade lesions are not overlooked (false negative rate).

The utility of EIS extends beyond its diagnostic capabilities. By improving the detection of high-grade cervical dysplasia, EIS supports the broader goals of cervical cancer prevention and early intervention. With more accurate identification of precancerous changes, clinicians can provide timely and appropriate treatment, potentially reducing the progression to cervical cancer. The review in hand examines the impact of EIS on cervical cancer screening, focusing on the performance of the method, the potential confounders that might mitigate its clinical utility and its future perspectives.

## Review

Overview of the technique

EIS is an analytical method that is used to measure the impedance, i.e., the resistance to current flow, in an electrochemical system across a range of frequencies. In the case of cervical EIS, the cervix with its different cellular layers, plays the role of the electrochemical system. This way, the technique highlights the electrochemical properties of the cervix and provides insights into its normal state and pathophysiology.

EIS involves applying a minimal alternating current (AC) signal through the tissue and measuring the resulting voltage using a small probe. The ratio of voltage to current equals the impedance, which can be expressed as a complex number with a real (resistive) and an imaginary (reactive) component [9]. The AC signal varies across a spectrum of frequencies (millihertz (mHz) to megahertz (MHz)). Thanks to this exact spectrum, it is possible to analyze different layers and structures within the cervix. Consequently, the technique can be used for purposes of tissue characterization (i.e., normal or abnormal depending on structure and cellularity), monitoring, and diagnosis (dysplasia, precancerous conditions, and cancer).

The advantages of the method are that it is non-invasive, provides real-time data on tissue health, yields results more quickly than a traditional biopsy (which constitutes the gold standard for diagnosis), and can be used in conjunction with other routine screening methods of the cervix (colposcopy, VIA) without demanding special preparation. On the other hand, the interpretation of EIS results requires sophisticated models and analytic techniques, whereas calibration and control are critical for acquiring accurate and reproducible results; otherwise, the ensuing variability may lead to variations in impedance measurements. The same may happen depending on individual patient and examiner differences.

The actual procedure of EIS is as follows: the patient is prepared for colposcopy as per usual practice. After the recognition of the cervix uteri, we apply 5% acetic acid in order to recognize possible epithelial lesions. Next, we perform a series of 10-12 measurements (which cover 90% of the cervical transformation zone) with ZedScan I, following the motif depicted on the device's screen. ZedScan I shows the results of the measurements as a series of colored spots, following the same motif as the examination. Then, the areas with the highest risk of HG-CIN are highlighted with different colors, according to the measurements. The regions with the highest probability are indicated with red color, whereas other possible regions are indicated with orange. The areas that are unlikely to foster HG-CIN are highlighted in green. If there is no indication for biopsy by ZedScan, biopsies are taken either based on the colposcopy assessment or randomly.

In order to specify the exact position of ZedScan-guided biopsy, the device is set in "single point mode", which collects and analyzes data of impedance as before, with the difference that this time the results are available in real-time. The probe is placed at the point that the operator judges to bear the highest probability for HG-CIN, based on the previous indication of the device. As soon as the device is ready, the operator performs the sampling. If the point is probable for HG-CIN, an orange light-emitting diode (LED) starts flashing at the probe, and a red circle appears on the device's screen. Consequently, the operator applies the biopsy forceps exactly on the reading point and performs the biopsy. In case the point does not satisfy the criteria for HG-CIN, a green LED flashes at the probe, and a green circle appears on the device's screen. If so, ZedScan should be applied on a neighboring point, and the procedure should be repeated until the device confirms the presence of HG-CIN or the operator decides to perform biopsies randomly or according to the colposcopy assessment. Each biopsy sample is placed in a different container before being sent for microscopy.

Material and methods

For this narrative review, we followed the checklist and rules mandated by Green et al. [10]. We conducted an electronic search of the English literature primarily in PubMed/Medline and adjunctively in Google Scholar and ResearchGate using the keywords "impedance" and "cervi*" from 2000 onwards. The references of key publications were also searched for potentially eligible studies. The search took place in April 2024. The inclusion criteria comprised studies that examined the clinical application of EIS in humans for detecting HG-CIN (CIN2 and CIN3). Studies not published in the English language, not performed in human tissues, addressing malignancies other than cervical dysplasia/neoplasia (i.e., melanoma, head and neck cancer, etc.), and referring to cervical applications other than dysplasia/neoplasia (i.e., parturition, preterm birth screening, etc.) were excluded. We included all types of clinical studies, prospective and retrospective. The presence of a comparator or control group (i.e., colposcopy only) was desirable but not mandatory for inclusion. Similarly, we focused on studies that mentioned at least one performance metric, but this was not mandatory in case the other eligibility criteria had been met.

Two authors separately extracted the following data for each included study: first author, year of publication, country/-ies of origin, doi number, (characteristics of the) implemented EIS platform, number of samplings per patient, study design, number of patients, presence of and number of patients in control group, and metrics used including sensitivity, specificity, positive predictive value (PPV), negative predictive value (NPV), and area under the receiver operating characteristic (ROC) curve (AUC). Regarding the latter, AUC ≤0.50 represented failure, 0.51-0.70 represented poor performance, 0.71-0.80 fair, 0.81-0.90 good, and 0.91-1.00 excellent performance. Wherever there was a p statistic available, p<0.05 was considered significant.

Results

**Figure 1:**
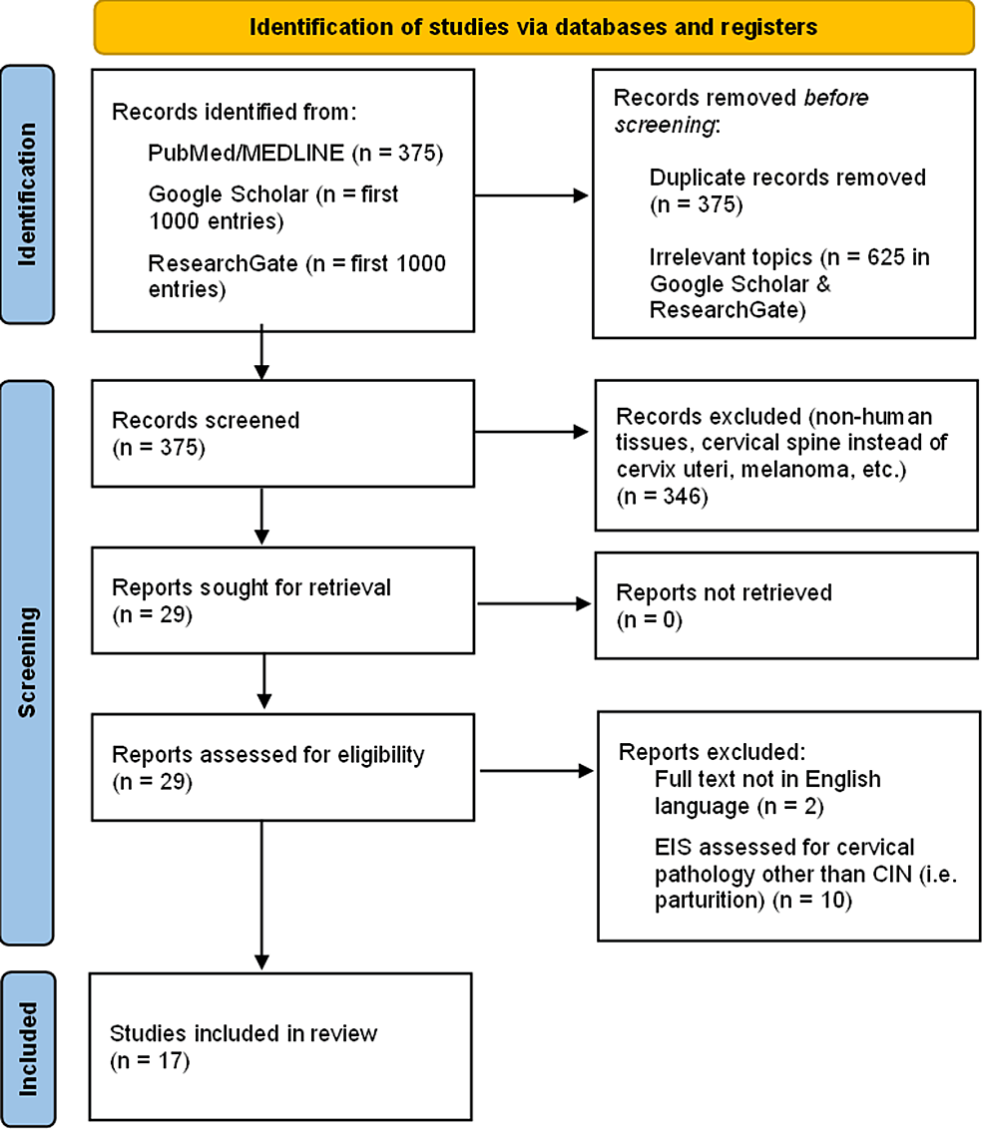
Study flowchart Preferred Reporting Items for Systematic Reviews and Meta-Analysis (PRISMA)-compliant flow diagram depicting the selection process of sources of evidence until the final study inclusion

The search in PubMed/Medline yielded 375 articles, the titles and abstracts of which were screened for relevance. Simultaneously, we searched through the first 1000 entries in Google Scholar and ResearchGate. There were no unique entries in the two latter databases that did not correspond to studies retrieved through PubMed in accordance with our inclusion and exclusion criteria; as such, duplicates were omitted. Eventually, we included 17 different papers in our analysis, for which we retrieved the full texts [11-27]. Figure 1 illustrates the flow diagram that we followed until the final study inclusion.

The studies spanned the period 2005-2023. Most publications came from the UK (nine studies) [11-13,15-17,20,25,26], followed by Greece (two studies) [24,27]. The other countries (Finland, India, Poland, Colombia, France, and Korea) had one contribution each. Different generations (which translates into different ranges of frequencies) of a four-electrode probe with sampling from multiple cervical sites (8-12) was the implemented method in all of the studies, except one that utilized a 17-electrode probe with single cervical sampling [23]. Most studies were single-center or oligo-centric, except one that was multi-centric and multinational; hence, it contained the largest number of participants (5257) [26]. Twelve studies were prospective observational [11-16,20-23,25,26], two were retrospective [17,18], and three were cross-sectional [19,24,27]. Two papers came from the same center and contained the same dataset of patients with different parameters examined [11,12]. Finally, two studies contained a control group against which the performance of EIS was compared [14,23], whereas another four studies compared the performance of EIS as an adjunct to colposcopy versus colposcopy alone [20,22,26,27].

Regarding the performance of EIS for detecting HG-CIN against normal tissue, sensitivity ranged from 0.387 to 1.000, specificity 0.060-0.947, PPV 0.321-1.000, and NPV 0-1.000, whereas the range for AUC was between 0.596 and 0.930. In the comparative study of Bergqvist et al., colposcopy combined with EIS outperformed colposcopy alone with regards to sensitivity (relative risk (RR) 1.16, 95% confidence interval (CI) 1.09 - 1.23, p=0.0007), whereas the opposite was true regarding specificity (RR 0.130, 95% CI 0.02 - 0.89, p 0.0033) [14]. The PPV was 1.00 for EIS and 0.86 for the control group, whereas NPV was 0 and 0.14, respectively. Similarly, in the large multinational study by Tidy and Brown, EIS held a sensitivity of 0.916 (vs. 0.741 in the control group, p<0.0001) and a maximum PPV of 0.9 [26]. Finally, our previous research showed that colposcopy + EIS had a sensitivity of 0.895 (vs. 0.855 in the control group), a specificity of 0.840 (vs. 0.970), a PPV of 0.944 (vs. 0.97), and an NPV of 0.724 (vs. 0.676) [24]. Interestingly, the AUC value of colposcopy + EIS was 0.87 (95% CI 0.78 - 0.96, p<0.001), whereas that of colposcopy alone was 0.89 (95% CI 0.81 - 0.97, p<0.001). Most importantly, though, the agreement rate with biopsy results was 0.792 in the EIS group vs. 0.743 in the control group (p<0.001). EIS performed better than colposcopy alone regarding underestimation (0.079 vs. 0.218) but somewhat worse regarding overestimation of premalignant lesions (0.129 vs. 0.004). Table 1 summarizes the basic features and the performance metrics reported by the included studies.

**Table 1:** Study characteristics Characteristics of the included studies and performance metrics. AUC - area under the (ROC) curve; CI - confidence interval; CIN - cervical intraepithelial neoplasia; EIS - electrical impedance spectroscopy; HG-CIN - high-grade cervical intraepithelial neoplasia; HSIL - high-grade squamous epithelial lesion; N - number of patients; N/A - not available; NPV - negative predictive value; NS - nonsignificant; PPV - positive predictive value; R - R parameter of Cole's equation; S - S parameter of Cole's equation.

First author	Year of publication	Country/-ies of origin	Platform	No. of samplings/pt	Study design	N (total)	N (EIS)	N (Control)	Performance metrics	Definition of high-grade cervical pathology	Sensitivity	Specificity	PPV	NPV	AUC		
Abdul et al. [11]	2005	UK	4-electrode probe	8	Prospective observational	176	176	N/A	-	CIN 2/3	0.74	0.53	0.6	0.67	0.88	-	-
Abdul et al. [12]	2006	UK	4-electrode probe, Mk III (2-1200 kHz)	8	Prospective observational	176	176	N/A	Per woman	CIN 2/3	0.74	0.53	0.6	0.67	0.880 (R)	0.830 (S)	0.652 (R/S)
Balasubramani et al. [13]	2009	UK	4-electrode probe, 76.3-625 kHz	12	Prospective observational	165	165	N/A	Total (pre acetic acid)	HG-CIN	0.788	0.73	-	-	0.8	-	-
									Per woman (pre acetic acid)	-	0.884	0.639	-	-	0.75	-	-
									Total (post acetic acid)	-	0.789	0.66	-	-	0.79	-	-
									Per woman (post acetic acid)	-	0.897	0.5	-	-	0.74	-	-
									p	-	-	-	-	-	0.98	-	-
Bergqvist et al. [14]	2023	Finland, UK	ZedScan	12-Oct	Prospective observational	1609	647	962	EIS	HSIL	1	0.06	1	0	-	-	-
									Control	-	0.86	0.46	0.86	0.14	-	-	-
									RR	-	1.16	0.13	-	-	-	-	-
									95% CI	-	1.090-1.23	0.020-0.890	-	-	-	-	-
									p	-	-	0.0033	-	-	-	-	-
Brown et al. [15]	2000	UK	4-electrode probe, Mk I (4.8-614 kHz)	8	Prospective observational	124	124	N/A	Per woman, R/S	CIN 2/3	0.75	0.71	0.89	0.45	0.819	-	-
Brown et al. [16]	2005	UK	4-electrode probe, Mk I (4.8-614 kHz)	8	Prospective observational	87	87	N/A	-	CIN 2/3	0.75	0.71	0.88	0.5	0.930 (R)	0.880 (S)	0.707 (R/S)
Brown et al. [17]	2020	UK	ZedScan	12	Retrospective	847	847	N/A	152 Hz	CIN2+	0.387	0.834	-	-	0.621	-	-
									1.22-2.44 kHz	-	0.452	0.701	-	-	0.596	-	-
Das et al. [18]	2015	India	Miniature planar electrode model 8W1E	26	Retrospective	150	150	N/A	N/A	-	-	-	-	-	-	-	-
Homola et al. [19]	2019	Poland	ZedScan	12	Cross-sectional	143	143	143	-	CIN2+	0.963	0.396	0.321	0.973	-	-	-
									95% CI	-	0.810-0.999	0.295-0.504	0.283-0.362	0.838-0.996	-	-	-
Macdonald et al. [20]	2017	UK	ZedScan I	N/A	Prospective observational	839	839	839	HPV16	CIN2+	0.956	-	-	-	-	-	-
									95% CI	-	0.906-0.982	-	-	-	-	-	-
									Control	-	0.896	-	-	-	-	-	-
									95% CI	-	0.801-0.916	-	-	-	-	-	-
									p	-	0.0171	-	-	-	-	-	-
									Non-HPV16	-	0.969	-	-	-	-	-	-
									95% CI	-	0.919-0.990	-	-	-	-	-	-
									Control	-	0.797	-	-	-	-	-	-
									95% CI	-	0.719-0.858	-	-	-	-	-	-
									p	-	<0.0001	-	-	-	-	-	-
Miranda et al. [21]	2012	Colombia	4-electrode probe, Mk III (9.6-614 kHz)	8	Prospective observational	56	56	N/A	m	-	0.66	0.85	-	-	0.78	-	-
									ρ0	-	0.73	0.8	-	-	0.81	-	-
Muszynski et al. [22]	2017	France, UK	ZedScan I	12-Oct	Prospective observational	91	91	91	EIS	HSIL	0.933	0.344	0.412	0.913	-	-	-
									95% CI	-	0.770-0.991	0.237-0.470	0.303-0.531	0.720-0.988	-	-	-
									Control	-	0.613	0.8	0.613	0.817	-	-	-
									95% CI	-	0.438-0.763	0.685-0.995	0.438-0.763	0.700-0.896	-	-	-
Oh et al. [23]	2021	Korea	17-electrode probe, 0.625-100 kHz	17	Prospective observational	123	69	54	CIN	CIN	0.943	0.84	-	-	0.9	-	-
Panagakis et al. [24]	2023	Greece	ZedScan I	12	Cross-sectional	101	46	55	EIS	HG-CIN	0.895	0.84	0.944	0.724	0.87	-	-
									95% CI	-	-	-	-	-	0.780-0.960	-	-
									p	-	-	-	-	-	<0.001	-	-
									Control	-	0.855	0.92	0.97	0.676	0.89	-	-
									95% CI	-	-	-	-	-	0.810-0.970	-	-
									p	-	-	-	-	-	<0.001	-	-
Tidy et al. [25]	2013	UK	4-electrode probe, 76.3-625 kHz	12	Prospective observational	474	196	196	Control	HG-CIN	0.885	0.385	0.535	0.808	-	-	-
									95% CI	-	0.799-0.944	0.294-0.483	0.450-0.618	0.675-0.904	-	-	-
									EIS (cutoff 0.768)	-	0.885	0.651	0.67	0.877	-	-	-
									p	-	NS	<0.0001	0.0006	NS	-	-	-
									EIS (cutoff 0.390)	-	0.966	0.385	0.556	0.933	-	-	-
									p	-	0.006	NS	NS	0.0094	-	-	-
									EIS (cutoff 0.568)	-	0.92	0.516	0.603	0.89	0.887	-	-
									p	-	NS	0.0076	NS	NS	-	-	-
Tidy et al. [26]	2022	UK	ZedScan	12-Aug	Prospective observational, multinational	5257	1392	5257	Control	HG-CIN	0.741	-	0.446	-	-	-	-
									EIS	-	0.916	-	0.9	-	-	-	-
									p	-	<0.0001	-	-	-	-	-	-
Tsampazis et al. [27]	2022	Greece	ZedScan	12	Cross-sectional	86	86	86	EIS	CIN2+	1	0.947	0.962	1	-	-	-
									Control	-	0.807	1	1	0.75	-	-	-

**Figure 2:**
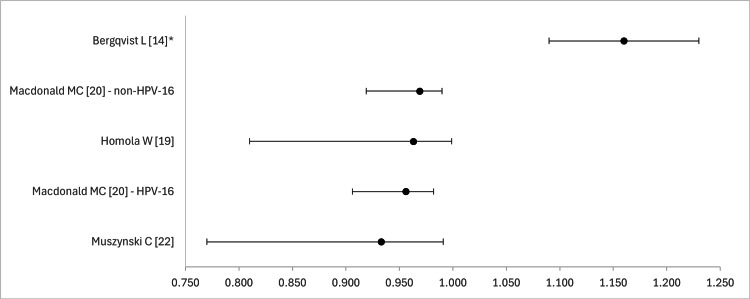
Forest plot that compares the sensitivity of EIS in studies reporting 95% CI values *relative risk compared to control EIS - electrical impedance spectroscopic

**Figure 3:**
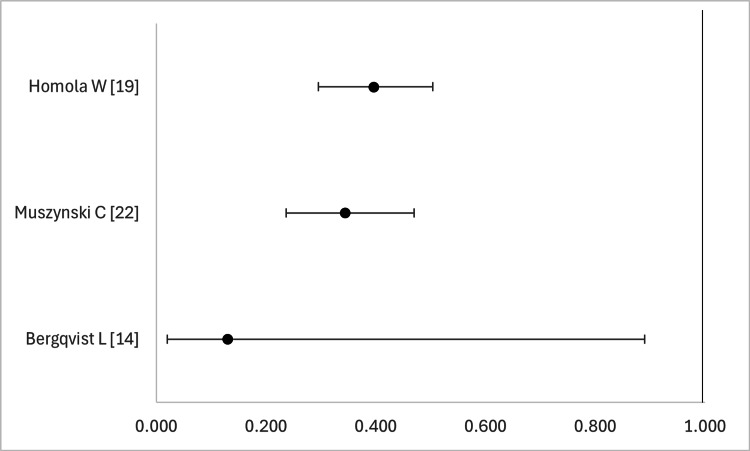
Forest plot that compares the specificity of EIS in studies reporting 95% CI values EIS - electrical impedance spectroscopic

Figures 2 and 3 depict forest plots that compare the sensitivities (Figure 2) and specificities (Figure 3) of the studies that mentioned 95% confidence intervals (95% CI). No more than 3 studies were available for PPV, NPV, and AUC, as such we did not procure similar diagrams for these metrics.

Several studies investigated potential confounders that might have mitigated the diagnostic accuracy of EIS. For instance, Balasubramani et al. explored the effect of acetic acid, the application of which constitutes a standard practice in colposcopy biopsy [13]. The authors concluded that there was no significant difference in the AUC before and after the application of acetic acid (p=0.98). Furthermore, in their 2020 publication, Brown et al. investigated the effect of low-frequency (512 Hz) and high-frequency (1.22-2.44 kHz) spectra and found a significant difference (p=0.0088) between the respective AUC curves in favor of the low-frequency spectrum [17]. They attributed this difference to the increases in extracellular volume and tissue homogeneity that occur in women who develop HG-CIN. Besides, in a series of two separate papers, Gandhi et al. highlighted the impact of different probe sizes and pregnancy status on the performance of EIS [28,29]. More specifically, they compared two probe sizes with different inter-electrode distances (small probe: 5mm, inter-electrode distance 2.2mm; small probe: 9mm, inter-electrode distance 3.9mm) and found a significantly higher resistivity of the cervix with the small probe over the frequency range 4-819 kHz (p<0.001) [28]. Additionally, the same group of investigators showed not only that the mean cervical stromal impedance (CSI) was higher in pregnant than non-pregnant women (p<0.01) but also that there were significant differences across different trimesters of pregnancy [29]. Another factor that may affect the measurements during EIS is the pressure applied to the probe, an issue that was raised by Gonzalez-Correa et al. after measuring a consistent proportional increase between pressure and resistivity in three different tissue types [30]. HPV genotype was another potentially confounding factor that was investigated by Macdonald et al. [20]. The authors showed that EIS is effective in detecting HG-CIN lesions irrespective of high-risk HPV genotype. Conversely, Miranda et al. showed that the number of different cellular layers that a tissue consists of might affect electrical bioimpedance, after using a physical-mathematic model to simulate the heterogeneous structure of the cervix and implementing the generalized effective medium theory of induced polarization (GEMTIP) [31]. These findings were challenged by the research of Vega et al., who applied the MOPET model (a GEMTIP-based model with more parameters) and subsequently analyzed the frequency spectra with Monte Carlo simulations (a probabilistic method for analyzing large numbers of data) [32]. The limitation of this study, as acknowledged by the investigators, is that its results apply to healthy tissue only. Table 2 summarizes all the documented factors that might affect EIS measurements based on evidence published so far.

**Table 2:** Potential confounders of EIS measurements Potential confounders of electrical impedance spectroscopy according to published studies * - evidence non-conclusive; EIS - electrical impedance spectroscopic

Potential confounders of EIS measurements
Frequency of EIS spectrum
Probe size
Pregnancy status and trimester
Pressure applied to the probe
Number of cellular layers on the cervical surface*

Discussion

In the present study, we compiled the available evidence on the clinical effectiveness of EIS as an adjunct to colposcopy for detecting high-grade CIN, with a focus on performance metrics. To our knowledge, this is the first attempt for a systematic recording of the utility of EIS dedicated to cervical pathology, given that relevant reviews in the past included either additional colposcopic adjuncts beyond EIS (i.e., DySIS) [7] or additional pathologies beyond CIN (i.e., melanoma, oral squamous cell carcinoma, etc.) [8].

The initial ascertainment is that the main stem of evidence derives from a specific group of investigators from Sheffield, UK, who were the first to conceptualize and subsequently popularize EIS as an adjunct for increasing the diagnostic accuracy of colposcopy regarding cervical pathology. Additionally, several other groups have confirmed the utility of EIS in independent studies. Besides, the chief investigators from the Sheffield group have orchestrated a large, multinational study across Europe and Israel that included more than 5000 women [26]. Nevertheless, in our opinion, more studies are needed in this regard to validate the reproducibility of the method in various contexts.

Secondly, although the majority of studies are prospective, none of them are randomized. Hence, one or more randomized trials are essential for establishing a causal association between the method and its improved outcomes as compared to colposcopy alone.

The evaluation of the method itself, based on the performance metrics, denotes that the pool of studies suffers with regard to homogeneity. For instance, an NPV ranging from 0% to 100% or an AUC ranging from 0.596 (which signifies poor performance) to 0.930 (which translates into excellent performance) are prohibitive in reaching a solid conclusion regarding the diagnostic effectiveness of EIS. Larger study populations and standardization of the method will contribute positively towards this direction. Additionally, the proposed confounders should be investigated further in future comprehensive research.

What would be some other means to optimize the outcomes of future research on EIS? The aforementioned study of Vega et al., where a Monte Carlo method was implemented for processing a large amount of data, paves the way in this respect [32]. Li et al. proposed a data-driven modeling approach based on logistic regression, a machine-learning classification method [33]. In other words, the advent of artificial intelligence might assist in managing a vast amount of measurements (aka *"*big data") in order to yield more reliable conclusions regarding the diagnostic validity of EIS. Another field of further investigation is warranted by the economic and survival benefit of the method, as this can be extrapolated by the biopsies spared by reducing false positive colposcopies as well as by the prompt diagnoses and interventions made in women conventionally diagnosed as false negatives, respectively [7].

As a narrative review, our study bears the main limitation of a non-comprehensive search strategy, although we need to acknowledge that the search was structured. In turn, this might have led to the incompleteness of the included studies and suboptimal objectivity. On the other hand, our review is one of the first of its kind, aiming to examine the current status, the areas of uncertainty or controversy, and the potential future directions of research on a promising, minimally-invasive, point-of-care method of prompt diagnosis and effective management, as is the case with electrical impedance spectroscopy.

## Conclusions

The need for enhanced diagnostics has led to the development of EIS as an adjunct to colposcopy for cervical intraepithelial neoplasia. According to the existing evidence, EIS can be a valuable tool for this purpose, particularly if one takes into consideration its relatively low cost and its minimally invasive nature. Nevertheless, larger-scale studies and variable settings are warranted to validate its clinical effectiveness. Additionally, potential confounders should be taken into account, both when designing and conducting these studies as well as when interpreting their results. The advent of artificial intelligence may constitute the next big step in integrating EIS into clinical practice, owing to its capability of processing big data.
